# B-lymphocytes as Key Players in Chemical-Induced Asthma

**DOI:** 10.1371/journal.pone.0083228

**Published:** 2013-12-13

**Authors:** Vanessa De Vooght, Vincent Carlier, Fien C. Devos, Steven Haenen, Erik Verbeken, Benoit Nemery, Peter H. M. Hoet, Jeroen A. J. Vanoirbeek

**Affiliations:** 1 Laboratory for Occupational and Environmental Toxicology, Leuven, Belgium; 2 Center for Molecular and Vascular Biology, Leuven, Belgium; 3 Translational Cell & Tissue Research, KU Leuven, Leuven, Belgium; Mie University Graduate School of Medicine, Japan

## Abstract

T-lymphocytes and B-lymphocytes are key players in allergic asthma, with B-lymphocytes producing antigen-specific immunoglobulins E (IgE). We used a mouse model of chemical-induced asthma and transferred B-lymphocytes from sensitized animals into naïve wild type mice, B-lymphocyte knock-out (B-KO) mice or severe combined immunodeficiency (SCID) mice. On days 1 and 8, BALB/c mice were dermally sensitized with 0.3% toluene diisocyanate (TDI) (20µl/ear). On day 15, mice were euthanized and the auricular lymph nodes isolated. B-lymphocytes (CD19^+^) were separated from the whole cell suspension and 175,000 cells were injected in the tail vein of naïve wild type, B-KO or SCID mice. Three days later, the mice received a single oropharyngeal challenge with 0.01% TDI (20µl) or vehicle (acetone/olive oil (AOO)) (controls). Airway reactivity to methacholine and total and differential cell counts in the bronchoalveolar lavage (BAL) fluid were measured 24 hours after challenge. B-lymphocytes of AOO or TDI-sensitized mice were characterized for the expression of surface markers and production of cytokines. We found that transfer of B-cells obtained from mice dermally sensitized to toluene diisocyanate (TDI) into naïve wild type mice, B-KO mice or SCID mice led, within three days, to an acute asthma-like phenotype after an airway challenge with TDI. This response was specific and independent of IgE. These B-lymphocytes showed antigen presenting capacities (CD80/CD86 and CD40) and consisted of B effector (Be)2- (IL-4) and Be1-lymphocytes (IFN-γ). The transferred B-lymphocytes were visualized near large airways, 24 hours after TDI challenge. Thus, B-lymphocytes can provoke an asthmatic response without the action of T-lymphocytes and without major involvement of IgE.

## Introduction

Many studies have demonstrated a crucial role for T-lymphocytes and the cytokines they produce in the development of allergic asthma [1]. In contrast, the exact role of B-lymphocytes in the development of asthma has been less well investigated [2], except for the well-known ability of B-lymphocytes to produce antigen-specific IgE antibodies after having been induced by Th2 cells to do so [3]. However, not all asthma is allergic (or atopic) asthma, and in a substantial proportion of asthmatics there seems to be little or no implication of specific IgE in the pathogenesis of the disease [4]. This is most notably the case in immunologically mediated occupational asthma (OA) caused by some sensitizing chemicals, such as diisocyanates [5]. Diisocyanates are low molecular weight chemicals widely used in industry for the production of e.g. polyurethane foams, vanish, paint, and isolation material [6,7]. They are an important cause of occupational asthma [5]. While high molecular weight agents, such as flour latex, enzymes, etc, can cause occupational asthma via the classical IgE mechanisms, sensitization to low-molecular-weight chemicals results from a response of the immune system to haptens conjugated with endogenous proteins. However, the exact pathways and mechanisms of sensitization to such chemicals and the pathogenesis of the subsequent respiratory reactions are much less well understood, as they seem to differ from those of the classic IgE-mediated asthma [8]. Lavaud et al. showed that showed that treatment of patients with severe occupational asthma due to low molecular weight agents, with the anti-IgE antibody omalizumab lowered the levels of total serum IgE and in most cases improved FEV1, but did not result in complete controlled asthma [9]. 

Recently, the pathophysiology of B-lymphocytes has received more interest and a number of new functions of B-lymphocytes have been identified, beyond the production of immunoglobulins. Clinical data show that B-lymphocyte depletion is an effective therapy for several T cell-mediated autoimmune diseases [10]. Lindell et al. showed that in asthma caused by cockroach allergen, B-lymphocytes also contribute to chronic allergic lung disease, possibly through antigen presentation, via promoting Th2 responses [2]. In addition, Harris et al. showed that B-lymphocytes can be subdivided into two subsets of effector B-lymphocytes (Be1 and Be2) depending on the cytokines they produce. Be1-lymphocytes (producing IFN-γ) regulate the differentiation of naïve Th-lymphocytes to Th1-lymphocytes, while Be2-lymphocytes (producing IL-4) regulate the differentiation to Th2-lymphocytes [11].

Our research group developed a robust mouse model for immunologically mediated chemical-induced asthma using a prototypical occupational asthmogen toluene diisocyanate (TDI) [12–20]. Because we were intrigued by the conundrum that isocyanate-induced asthma has many features of allergic asthma, both in humans and in mouse models, and yet does not appear to depend on the presence of (humoral) IgE antibodies in our model, we set out to investigate the role, if any, of B-lymphocytes in our mouse model. To achieve this, we characterized the profile of B-lymphocytes after dermal sensitization to TDI, on the one hand, and we performed adoptive transfer experiments using physiologically relevant amounts (175,000) of B-lymphocytes obtained from TDI-sensitized mice into naïve wild type mice, B-KO mice and severe combined immunodeficiency (SCID), which are mice deficient in T- and B-lymphocytes. 

We found that B-lymphocytes may play an important primary role in asthma, without help from T-lymphocytes.

## Materials and Methods

### Reagents

Toluene-2,4-diisocyanate (98 %; Fluka, CAS 584-84-9), trimellitic anhydride (97 %, CAS 552-30-7), acetyl-β-methylcholine (methacholine), acetone, phorbol myristate acetate (PMA, CAS 16561-29-8) and Ca^2+^ ionophore (CAS 56092-82-1) were obtained from Sigma-Aldrich (Bornem, Belgium). Pentobarbital (Nembutal®) was obtained from Sanofi Santé Animale (CEVA, Brussels, Belgium) and Isoflurane (Forene®) from Abbott Laboratories (S.A. Abbott N.V., Ottignies, Belgium). The vehicle (acetone/olive oil, AOO) used to dissolve TDI consisted of a mixture of 2 volumes of acetone and 3 volumes of olive oil (Selection de Almazara, Carbonell, Madrid, Spain) for the dermal sensitization, and 1 volume of acetone and 4 volumes of olive oil for the oropharyngeal challenge. Concentrations of TDI are given as percent (v/v) in AOO, while TMA concentrations are given as percent (w/v). 

### Animals

Male wild type BALB/c mice were obtained from Harlan (Horst, The Netherlands). Male Jh mice (BALB/c background), which are deficient in B-lymphocytes (labeled as B-KO mice, hereafter) and C.B-17 SCID mice (BALB/c background), which are deficient in T-lymphocytes and B-lymphocytes, were obtained from Taconic (Ejby, Denmark). All mice were approximately 20 g and 6 weeks old. The mice were housed in a conventional animal house in filter top cages with 12-h dark/light cycles and received lightly acidified water and pelleted food (Trouw Nutrition, Gent, Belgium) ad libitum. 

### Experimental setup

All experimental procedures were approved by the KU Leuven Ethical Committee for Animal Experiments.

The experimental protocols were based on our previously published protocol of chemical-induced asthma in which mice are first sensitized by receiving dermal applications of the test chemical on days 1 and 8 and then challenged via the airways with a lower concentration of the test chemical on day 15, with the responses (airway reactivity to methacholine, lung inflammation, immunologic readouts) being assessed 24 hours after challenge [14,19,20].

This complete protocol was applied in B-KO mice: on days 1 and 8, B-KO mice received dermal applications of 0.3 % TDI or vehicle (AOO) on the dorsum of both ears (20 µl/ear). On day 15, they received an oropharyngeal challenge with 0.01% TDI or vehicle [19]. Each treatment group consisted of 8 to 9 animals. Mice were euthanized 24 hours after the challenge, by intraperitoneal injection of pentobarbital (90 mg/kg).

In the adoptive transfer experiments, a similar protocol was used, except for the fact that sensitization and challenge portions took place in donor (D) and recipient (R) mice, respectively, with the cells (or serum) being obtained and transferred on day 15 and the challenge being done 3 days later. 

Thus, on days 1 and 8, the donor (D) animals received dermal applications of 0.3 % TDI, 5 % trimellitic anhydride (TMA) or the vehicle (20 µl/ear). On day 15, these mice were euthanized, auricular lymph nodes were pooled and the spleen was dissected. Cell suspensions were obtained by pressing the lymph nodes or spleen through a cell strainer (100 µm) (BD Biosciences, Erembodegem, Belgium) and rinsing with 10 ml buffer (MACS BSA Stock solution diluted 1:20 with autoMACS Rinsing Solution (Miltenyi Biotec, Utrecht, The Netherlands)). Cells were centrifuged (1000 g, 4 °C, 10 min) and counted using a Bürker hemocytometer. CD19^+^ B-lymphocytes were isolated with CD19^+^ microbeads (Miltenyi Biotec) according to manufacturer’s instructions, using pre-separation filters, LS columns and the QuadroMACS (Miltenyi Biotec). CD19^+^ B-lymphocytes or lymph node cells without B-lymphocytes were resuspended in HBSS^-^ buffer (Invitrogen, Merelbeke, Belgium) and 175,000 cells or 325,000 cells, respectively, were injected, in a total volume of 250 µl, intravenously in the tail vein of naïve recipient (R) mice. In a separate experiment, on day 15, mice were euthanized and blood was obtained via the retro-orbital plexus. The blood was centrifuged (14,000 g, 10 min) and 100 µl serum (D1: 1537 ± 702 ng/ml IgE as described in [21]) in a total volume of 250 µl (dissolved in HBSS^-^) were transferred in naïve wild type BALB/c mice. 

Three days after the transfer of B-lymphocytes, lymph node cells without B-lymphocytes or serum, recipient mice received an oropharyngeal challenge with 0.01 % TDI, 0.05 % TMA or vehicle. Each treatment group consisted of 3 to 10 animals. Mice were euthanized 24 hours after the challenge.

Experimental groups are labeled as follows: D_TDI_R_Veh_, D_TDI_R_TDI_, D_TMA_R_TMA_, D_TMA_R_Veh_ and D_TDI_R_TMA_, with D indicating the dermal treatment (sensitization) received by donor (D) animals on days 1 and 8, i.e. either TDI (D_TDI_) or TMA (D_TMA_), and R indicating the type of challenge received by the naïve recipient (R) animals three days after having received the B-lymphocytes, i.e. vehicle (R_Veh_), TDI (R_TDI_) or TMA (R_TMA_). 

### Airway hyperreactivity (AHR)

Twenty four hours after the challenge, reactivity to methacholine was assessed invasively using a forced oscillation technique (FlexiVent, SCIREQ, Montreal, Canada) [22]. As previously described, airway resistance (R) was measured using a “snapshot” protocol. For each mouse, R was plotted against methacholine concentration (0 mg/ml tot 10 mg/ml) and the AUC was calculated to perform statistical analysis [22].

### Bronchoalveolar lavage and lung histology

After measuring AHR, mice were deeply anesthetized by an intraperitoneal injection of pentobarbital (90 mg/kg body weight). Blood was taken from the retro-orbital plexus, centrifuged (14000 g, 10 min) and serum samples were stored for further analyses. The lungs were lavaged, in situ, three times with 0.7 ml sterile saline (0.9 % NaCl), and the recovered fluid was pooled. Cells were counted using a Bürker hemocytometer (total cells) and the BAL fluid was centrifuged (1000 g, 10 min). For differential cell counts, 250 µl of the resuspended cells (100,000 cells/ml) were spun (300 g, 6 min) (Cytospin 3, Shandon, TechGen, Zellik, Belgium) onto microscope slides, air-dried and stained (Diff-Quik® method, Medical Diagnostics, Düdingen, Germany). For each sample, 200 cells were counted for the number of macrophages, eosinophils, neutrophils and lymphocytes.

After BAL fluid collection, the lungs were instilled with 4% formaldehyde until full inflation of all lobes, as judged visually. Evaluation of lung injury on slides stained by hematoxylin and eosin was performed by an experienced pathologist who was blinded to the treatment given to the mice (AxioPlan microscope, objective Pan-Apochromat x40 with numeral aperture 0.95, Carl Zeiss, Zaventem, Belgium). Pictures were taken with an AxioCam HRc camera and acquisition was performed with AxioVision software 4.8 (Carl Zeiss, Zaventem, Belgium).

### Total serum IgE

The OptEIA Mouse IgE set from Pharmingen (BDBiosciences) was used to measure total serum IgE (diluted 1/70). Measurements were performed according to the manufacturer’s instructions.

### Surface marker expression on B-lymphocytes

On day 15, wild type BALB/c mice dermally sensitized with TDI (or AOO) were euthanized and auricular lymph nodes were dissected and cell suspensions were obtained as described above. 500,000 cells were stained with anti-CD19 (PerCP-Cy5.5, BD Biosciences, Erembodegem, Belgium), anti-major histocompatibility complex II (MHCII, PE), anti-IgD (PE), anti-CD23 (FITC), anti-CD5 (FITC), anti-CD40 (FITC), anti-CD86 (PE) and anti-CD80 (PE) labeled antibodies, according to standard procedures, and with control samples being labelled with isotype match control antibodies (BD Biosciences, Erembodegem, Belgium). Flow cytometry (FACS Calibur, BD Biosciences, Erembodegem, Belgium) was performed using at least 10^5^ cells.

### Intracellular cytokine staining

On day 15, wild type BALB/c mice sensitized with TDI or AOO were euthanized and auricular lymph nodes were dissected and cell suspensions were obtained as described above. Intracellular cytokine staining was performed according to manufacturer’s instructions (BD Biosciences, Erembodegem, Belgium). Briefly, lymphocytes were restimulated in vitro with PMA (5 ng/ml) and Ca^2+^ ionophore (500 ng/ml). BD GolgiStop^TM^ containing monensin (BD Biosciences, Erembodegem, Belgium) was added one hour after the culture was initiated. Lymphocytes were collected 5 hours later and stained for anti-CD19 surface marker (APC-Cy7). Afterwards, cells were fixed and permeabilized, and incubated with anti-IFN-γ (PE-Cy7), anti-IL-4 (APC) and anti-IL-10 (PE) labeled antibodies. Flow cytometry (FacsArray, BD Biosciences, Erembodegem, Belgium) was performed using at least 10^5^ cells. 

### B-cell homing study with SNARF-1 labeling

On day 15, wild type BALB/c mice sensitized with TDI were euthanized; auricular lymph nodes were dissected and B-lymphocytes were isolated as mentioned above. Freshly isolated B-lymphocytes were incubated in PBS^-^ (Invitrogen, Merelbeke, Belgium) with 125 nM of the succinimidyl ester of SNARF-1 carboxylic acid acetate (Invitrogen, Merelbeke, Belgium) for 15 minutes at 37 °C. Afterwards, the cells were washed twice with RPMI-1640 medium and resuspended in HBSS^-^ to be transferred into naïve wild type BALB/c mice. 5x10^6^ labeled B-lymphocytes were transferred. Three days after transferring the labeled B-lymphocytes, mice were challenged with 0.01 % TDI or vehicle and 24 hours later lungs were dissected after perfusion of the mice with NaCl. The distribution of transferred B-lymphocytes was investigated using fluorescence microscopy (Olympus BX61, objective x40 oil with numeral aperture 1.30) on cryostat sections (sagittal axis, 10 µm sections) of the lung mounted in ProLong^®^ Gold antifade reagent with DAPI (Invitrogen, Merelbeke, Belgium). Photographs were taken with a digital color camera UC30 3Mpixel (Olympus, Aartselaar, Belgium) and acquisition was performed with Cell F Software (Olympus, Aarstselaar, Belgium).

### Statistical analysis

Normality of distribution of the data was assessed by the D’Agostino & Pearson omnibus normality test. All data are presented as means or means and SEM. AHR and the surface markers were analyzed using an unpaired t-test, whereas the airway inflammation and the intracellular cytokine stainings were analyzed using a nonparametric Mann-Whitney test (Graphpad Prism 4.01, Graphpad Software Inc, San Diego, USA). A level of p < 0.05 (two tailed) was considered significant.

## Results

### Characterization of B-lymphocytes and serum from donor mice

**Figure 1:**
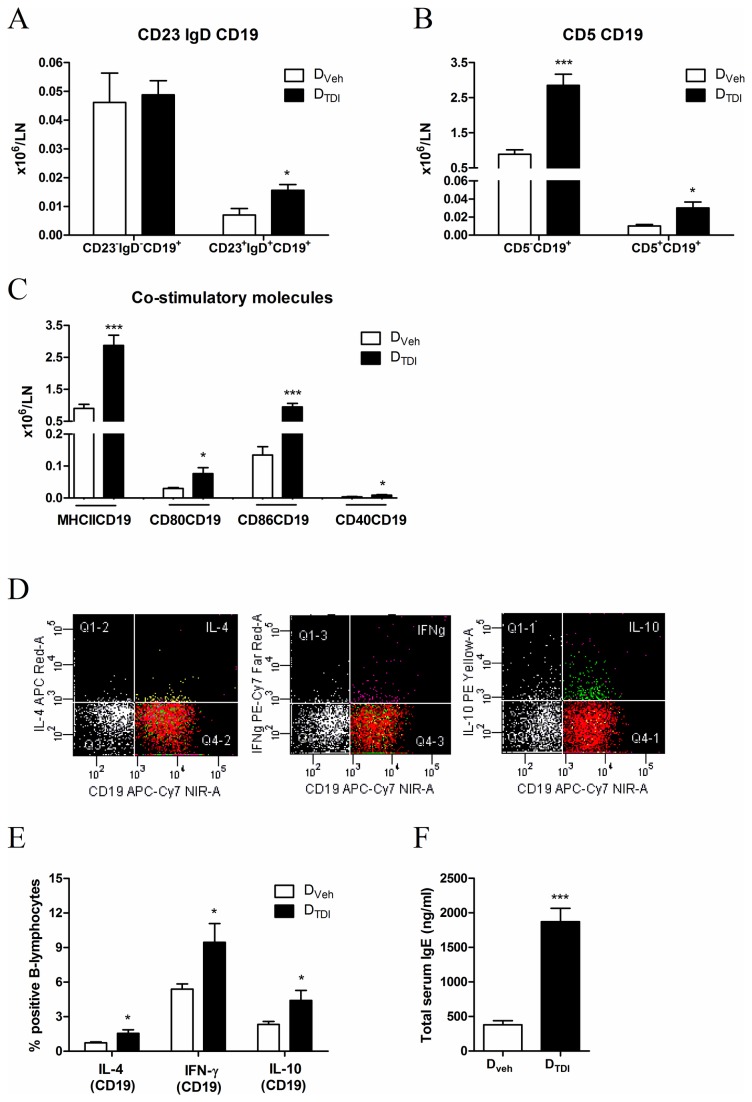
Total serum IgE in donor mice along with surface markers and cytokine production by B-lymphocytes of the auricular lymph nodes. Serum and lymphocytes from the auricular lymph nodes were obtained of mice dermally treated with vehicle (D_Veh_) or TDI (D_TDI_). The lymphocytes were stained with anti-CD19 to identify B lineage cells and for different surface markers and co-stimulatory molecules. CD19^+^-lymphocytes were co-stained with anti-CD23 and anti-IgD to distinguish between follicular and marginal zone B-lymphocytes (A); co-stained with CD5 to distinguish between B1- (B1a) and B2-lymphocytes (B) and co-stained with MHCII, CD86, CD80 and CD40 to characterize the antigen presentation capacity of the B-lymphocytes (C). Lymphocytes of auricular lymph nodes were cultured in vitro for 5 hours with PMA, Ca^2+^ ionophore and monensin. Anti-CD19 was used to identify B lineage cells and the percentage B-lymphocytes staining intracellularly for the cytokines IL-4, IFN-γ and IL-10 was assessed. Figure 1 D shows representative dot plots of intracellular cytokine expression in B-lymphocytes from TDI-sensitized mice. The percentage of the total B-lymphocytes expressing cytokines is quantified in graph E. In the serum of D_veh_ or D_TDI_ mice, total IgE levels were measured (F). Data are presented as means ± SEM, n = 4-5, * p < 0.05 and *** p < 0.001.

Blood was collected from vehicle and TDI-treated mice. Figure 1 F shows significantly increased levels of total serum IgE in TDI-sensitized mice compared to vehicle treated mice.

We characterized the B-lymphocytes isolated from the auricular lymph nodes of TDI or vehicle treated mice (Figure 1). On the basis of different surface markers we distinguished several B-lymphocyte subpopulations. Sensitization with TDI resulted in a significantly increased number of follicular B-lymphocytes (CD23^+^IgD^+^CD19^+^) (Figure 1 A) as well as increases in CD5^+^ B-lymphocytes (B1a) and CD5^-^ B-lymphocytes (B1b and B2) (Figure 1 B) in the auricular lymph nodes. All B-lymphocytes expressed MHCII, independently of TDI or AOO treatment. Co-stimulatory molecules CD86, CD80 (activation of T-lymphocytes) and CD40 (activation of B-lymphocytes) were upregulated in B-lymphocytes from TDI-treated mice (D_TDI_) compared to control vehicle-treated mice (D_Veh_) (Figure 1 C).

Cytokine production was assessed by stimulating cultured lymph node cells for 5 hours with PMA and Ca^2+^ ionophore in the presence of monensin. CD19^+^ B-lymphocytes from TDI-sensitized mice thus produced significantly higher levels of IL-4, IFN-γ and IL-10 than B-lymphocytes from AOO-treated mice (Figure 1 E). The FACS plots (Figure 1 D) showed a mixed B effector (Be)1 (IFN-γ) - Be2 (IL-4) response. 

### Adoptive transfer experiments into naïve wild type BALB/c mice

**Figure 2:**
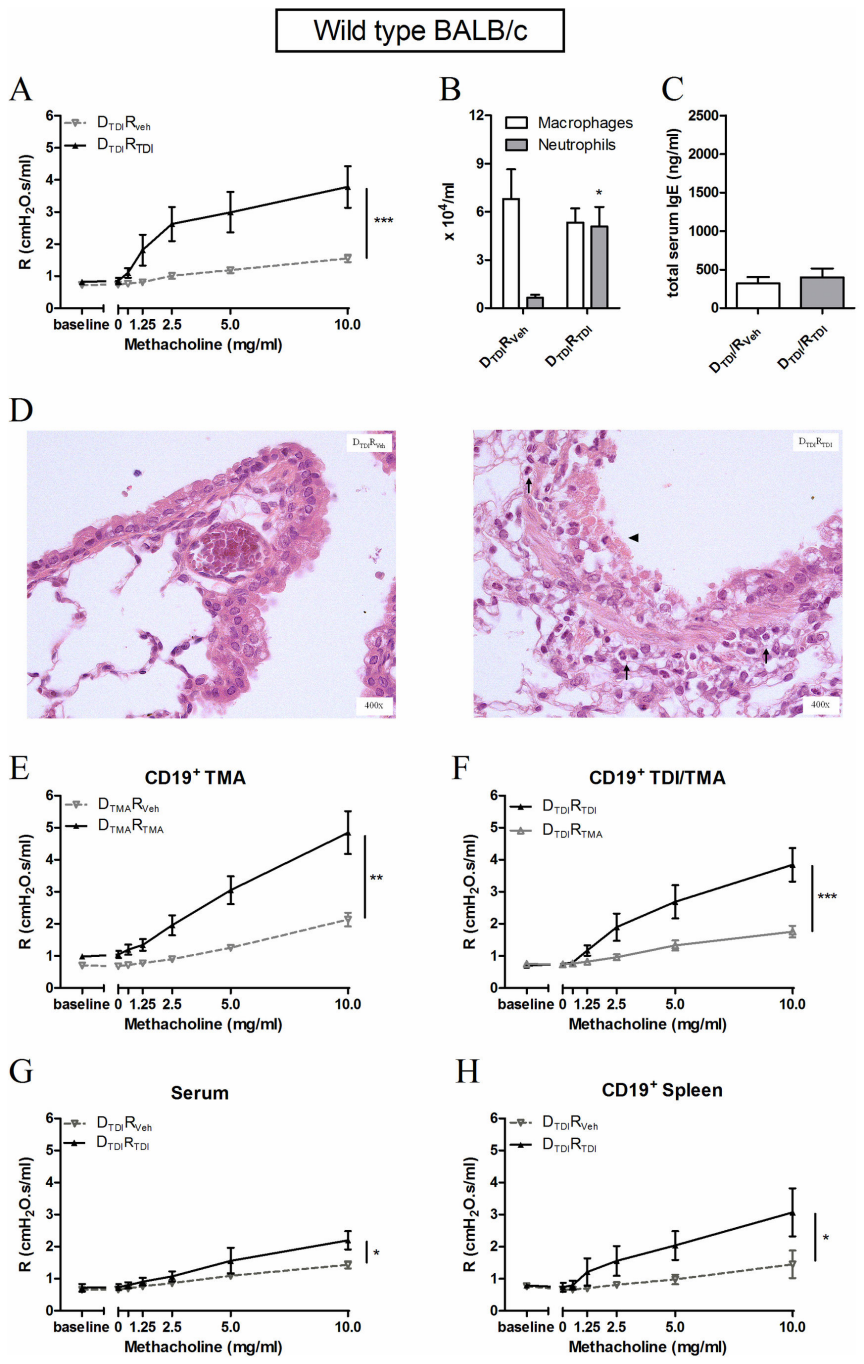
Production of an asthma-like response in naïve wild type BALB/c mice after having received B-lymphocytes. Experimental groups are D_TDI_R_Veh_, D_TDI_R_TDI_, D_TMA_R_Veh_, D_TMA_R_TMA_ and D_TDI_R_TMA_. D represents donor (D) animals that received dermal applications of TDI (D_TDI_) or TMA (D_TMA_) on days 1 and 8. Their B-lymphocytes were transferred into naïve recipient (R) mice which received a challenge with vehicle (R_Veh_), TDI (R_TDI_) or TMA (R_TMA_) three days after the transfer. Airway resistance (R), after increasing concentrations of methacholine (0-10 mg/ml), was measured using a forced oscillation technique, 22 hours after the challenge. Figure 2 A reflects the airway hyperreactivity (AHR) to increasing concentrations of methacholine after transferring B-lymphocytes of auricular lymph nodes into wild type BALB/c mice. Macrophages and neutrophils were identified in the BAL fluid (B) and in lung tissue (D) 24 hours after the challenge. Total serum IgE was measured (C). Figure 2 E, F, G and H represent AHR of the experiment assessing the transfer of the B-lymphocytes of TMA sensitized mice, the specificity of the B-lymphocytes to TDI, the transfer of serum and the transfer of B-lymphocytes of the spleen, respectively. Data are presented mean ± SEM, n = 4-10 per group, * p < 0.05, ** p < 0.01 and *** p < 0.001 compared with the DTDIRVeh group (A, B, C, G and H) and with DTMARTMA (E). Symbols: (↑) inflammation and (▲) epithelial damage.

Freshly isolated B-lymphocytes were transferred into naïve wild type BALB/c mice in order to assess the specific role of B-lymphocytes (Figure 2). 

In a first series, B-lymphocytes were isolated from the auricular lymph nodes of TDI-sensitized wild type BALB/c mice and then transferred into naïve wild type BALB/c mice. Three days later, the mice were challenged with TDI and this resulted in an increase in airway reactivity (Figure 2 A) and airway inflammation (Figure 2 B), but not in total serum IgE levels, 24h later (Figure 2 -C). Histology revealed an influx of polymorphonuclear leukocytes and epithelial damage in the D_TDI_R_TDI_ group (Figure 2 D). We also transferred the reciprocal lymphocyte population (without B-lymphocytes) into naïve mice, but this did not alter airway reactivity or produce airway inflammation after TDI challenge compared to their control mice (data not shown). 

In a second series of experiments, we assessed the specificity of the B-lymphocytes for TDI. Here, we show that transferring B-lymphocytes from trimellitic anhydride (TMA), another known potent chemical respiratory sensitizer, mice into naive mice, followed by a TMA challenge 3 days later results in AHR (Figure 2 E) and airway inflammation (data not shown), indicating that the transfer model also works with other chemical sensitizers. Next, mice that received B-lymphocytes obtained from TDI-sensitized mice were challenged with trimellitic anhydride (TMA). This yielded no increase in AHR (Figure 2 F) and no airway inflammation (data not shown), indicating that the responses with TDI sensitization followed by TDI challenge were indeed linked to recognition of TDI by the B-lymphocytes. 

In a third series, the involvement of immunoglobulins possibly secreted by the transferred B-lymphocytes was explored. Transferring serum obtained from TDI-sensitized mice into naïve mice followed by a TDI challenge resulted in limited airway inflammation (data not shown) and a less pronounced, although significant, airway hyperreactivity (AHR) after TDI challenge (Figure 2 G). 

Finally, B-lymphocytes isolated from the spleen of TDI-sensitized mice and transferred into naïve wild type BALB/c mice induced AHR (Figure 2 H) after challenging the mice with TDI, but no significant lung inflammation was found (data not shown).

### Adoptive transfer experiments into naïve immune-compromised BALB/c mice

B-KO mice were used to confirm a role for B-lymphocytes in chemical-induced asthma. 

First, our mouse model using two dermal applications of TDI and one oropharyngeal challenge with TDI was tested in the B-KO mice. In these animals (as well as in appropriate controls), AHR remained low, no airway inflammation was observed and no increased levels of total serum IgE were found (Veh: 221.4 ± 66.5 ng/ml vs. TDI: 264.9 ± 105.7 ng/ml) thus confirming the importance of B-lymphocytes in our model (data not shown). 

**Figure 3:**
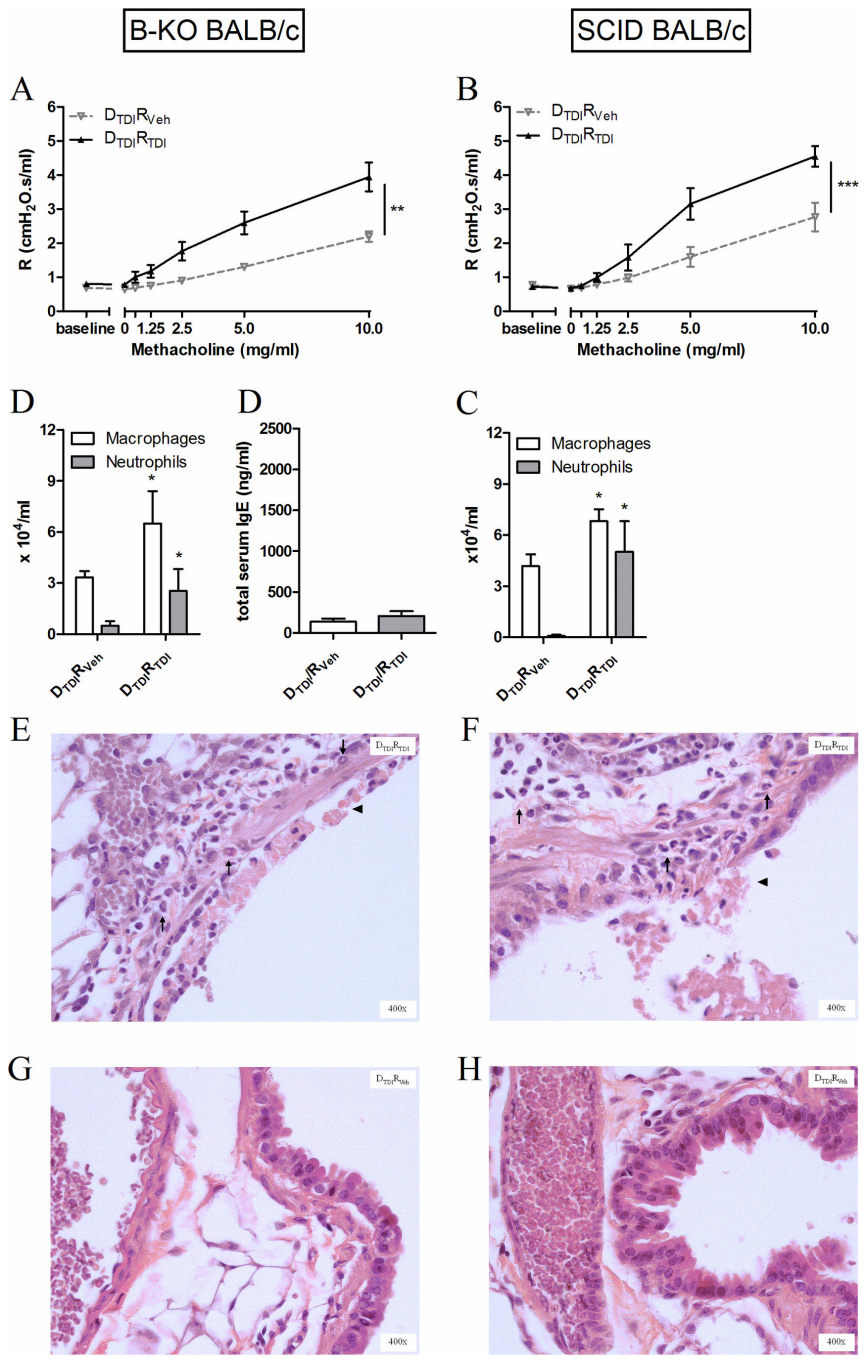
Transferring B-lymphocytes leads to an asthma-like response after TDI challenge in B-KO and SCID BALB/c mice. Airway methacholine reactivity was measured after transferring B-lymphocytes in B-KO (A) or SCID (F) mice. Macrophages and neutrophils were identified in the BAL fluid (B and G) and in lung tissue (D, E and H, I) 24 hours after the challenge. Total IgE was assessed in serum of B KO mice. Experimental groups for the adoptive transfer setup are identical to those of Figure 2 (DTDIRVeh and DTDIRTDI). Data are presented as means ± SEM, n = 5-8 per group, * p < 0.05, ** p < 0.01, *** p < 0.001 compared to the DTDIRVeh group. Symbols: (↑) inflammation and (▲) epithelial damage.

Second, B-lymphocytes were isolated from the lymph nodes of TDI-sensitized BALB/c mice and transferred into naïve B-KO mice. The adoptive transfer of B-lymphocytes now resulted in airway hyperreactivity after TDI challenge (Figure 3 A), significant increases in neutrophils and macrophages the BAL fluid (Figure 3 B), but no increased level of total serum IgE (Figure 3 C). Histological analysis of these lungs confirmed the airway inflammation and epithelial damage (Figure 3 D-E).

Third, B-lymphocytes from lymph nodes of TDI-sensitized mice were transferred into naïve SCID mice. Previously, we had shown that SCID mice are not able to develop an asthma-like response after dermal sensitization and challenge with TDI [15]. However, after transferring B-lymphocytes obtained from TDI-sensitized mice into naïve SCID mice a significant increase in airway reactivity (Figure 3 F) and airway inflammation (Figure 3 G) was found after TDI challenge. The influx of neutrophils in the lungs was confirmed on histology (Figure 3 H-I). 

### B-cell homing in the lung after adoptive transfer into naïve wild type BALB/c mice

**Figure 4:**
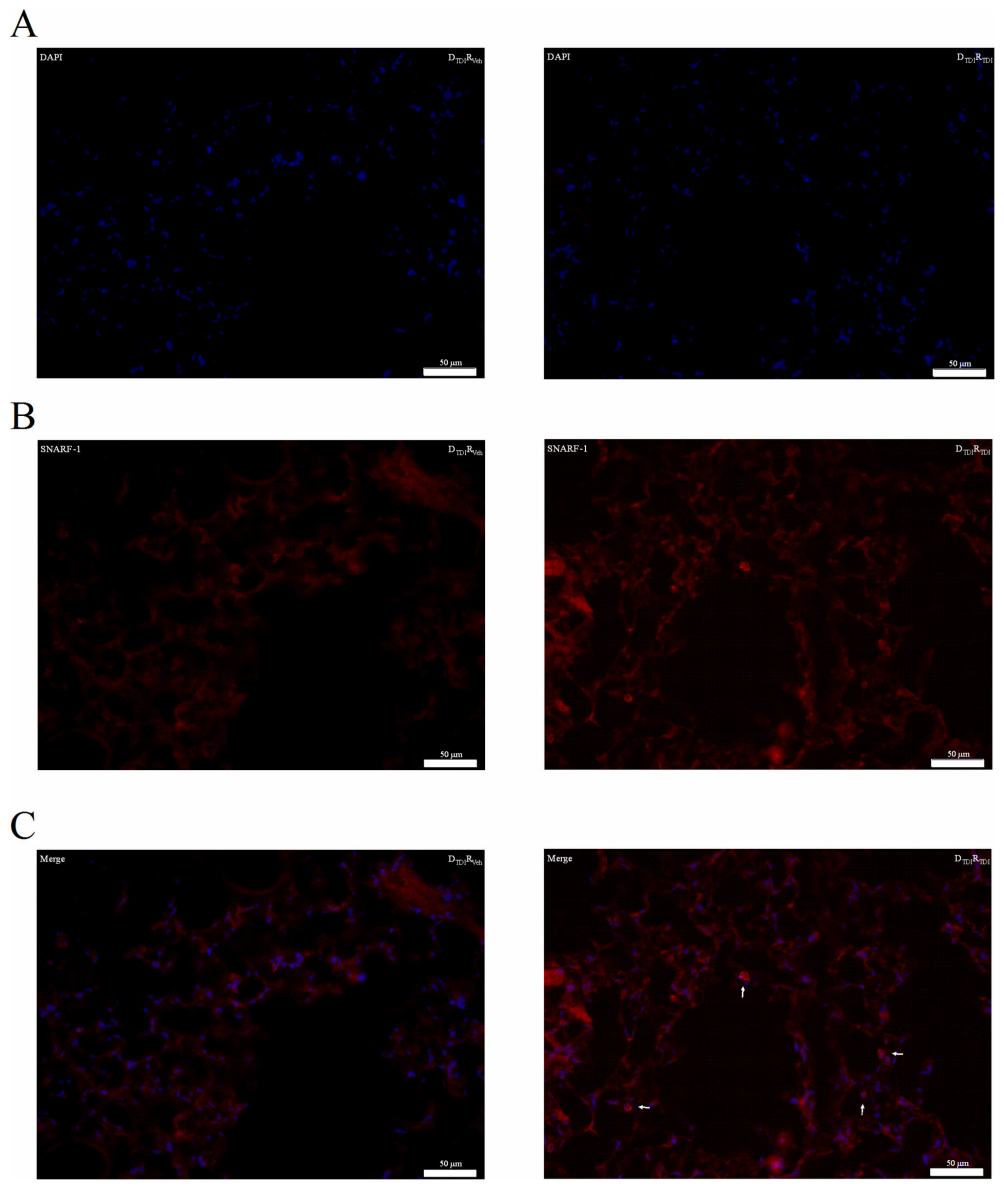
Transferred B-lymphocytes are present in the lungs of TDI challenged wild type BALB/c mice. Freshly isolated B-lymphocytes of the auricular lymph nodes of TDI-sensitized mice were labeled with DAPI and SNARF-1 carboxylic acid acetate and transferred into naïve wild type BALB/c mice. 5x106 labeled B-lymphocytes were transferred. Three days after the transfer mice were challenged with TDI and cryostat sections were made. Experimental groups for the adoptive transfer setup are identical to those of Figure 2 (DTDIRVeh and DTDIRTDI). Figure C shows the merged picture of the DAPI (A) and SNARF-1 (B) staining.

In figure 4, freshly isolated and labeled (SNARF-1) B-lymphocytes obtained from TDI-sensitized mice were transferred into naïve wild type mice to visualize their presence in the lung. SNARF-1 positive cells were found close to the airways of mice challenged with TDI and this was not the case in mice challenged with vehicle (Figure 4 B-C).

## Discussion

We investigated the role of B-lymphocytes in the development of non-atopic asthma using an established mouse model of chemical-induced asthma [13–15,18–20]. The main findings of this study were that B-lymphocytes play an important role in the induction of AHR and airway inflammation, even without the presence of T-lymphocytes. Furthermore, B-lymphocytes of TDI-sensitized mice were shown to produce cytokines that reflect a mixed Be1-Be2 response and express surface markers characteristic of antigen presentation capacity.

Studies on B-lymphocytes and their role in the immune response and more specifically in asthma have almost exclusively focused on their implication in the humoral response, i.e. the production of antigen-specific IgE antibodies. Recently, there has been growing appreciation that B-lymphocytes also play more central roles in orchestrating immune responses [23–25]. The role of B-lymphocytes in cellular immune responses has received renewed interest due to clinical data showing that B-lymphocyte depletion is an effective therapy for several T-cell mediated autoimmune diseases, while the therapy does not necessarily correlate with changes in the circulating autoantibodies [10]. In low molecular weight induced asthma, specific IgE antibodies are frequently not present, which suggests that non-IgE mediated mechanisms are involved in the pathogenesis [5].

Our data show that transferring B-lymphocytes from TDI-sensitized mice into naïve wild type mice resulted in an asthma-like response after a sensitizer-specific challenge. The protocol for the adoptive transfer of lymphocytes, designed and optimized previously, is unique because only low, physiologically relevant, quantities of lymphocytes are sufficient to obtain the desired response [21]. An amount of only 175,000 B-lymphocytes was enough to passively transfer TDI sensitization and develop an asthma-like response in naïve mice after TDI challenge. This is in contrast with other studies that transfer millions of B-lymphocytes [26–28]. In our experiment designed to study the homing of B-lymphocyte after their transfer, we injected a higher quantity, i.e. 5,000,000 labeled B-lymphocytes, in order to increase the chance of detecting labeled cells in the histological sections of the lung, and this proved successful. 

B-lymphocytes were isolated using CD19^+^ magnetic beads. CD19 is a surface glycoprotein expressed by early pre-B-lymphocytes and throughout B-lymphocyte development, but it is not present on plasma cells, indicating that no immunoglobulin producing cells were transferred [29,30]. To verify this, we transferred serum from TDI-sensitized mice into wild type naïve mice. Although our data showed limited airway inflammation and even to a lesser extent AHR after TDI challenge, this was minor compared to the results obtained after transferring B-lymphocytes, thus suggesting that antibodies are not sufficient to induce the response and that antibody-independent mechanism of B-lymphocytes can lead to an “allergic” response. This was also confirmed by the fact that we found no increases in total serum IgE levels in the wild type mice that received B-lymphocytes. The purity of the isolated B-lymphocytes was tested several times by FACS. The combined impurity (CD3^+^, CD4^+^, CD8^+^ and CD25^+^) was always less than 5% (data not shown), i.e. fewer than 10,000 cells. We do admit that the presence of T-lymphocytes and dentritic cells in this cell population might play a limited role in the response we find. 

In two separate experiments we also tested the specificity of our response, which represents an essential prerequisite for an adaptive immune response. In a first experiment we showed that the D_TDI_R_Veh_ group (transfer of TDI-sensitized B-lymphocytes followed by challenge with AOO) and the D_Veh_R_TDI_ group (transfer of AOO-treated B-lymphocytes followed by challenge with TDI, data not shown) showed neither increased AHR nor airway inflammation, the latter group proving (again) that the response observed after TDI challenge did not simply result from irritation. In a second experiment, naïve mice were transferred with TDI-sensitized B-lymphocytes and received a challenge with trimellitic anhydride, also a known respiratory sensitizer [14]. These mice showed no AHR and almost no inflammation in BAL compared to D_TDI_R_TDI_ mice, suggesting a TDI-specific asthmatic response triggered by the transferred B-lymphocytes.

Previously, Lindell et al. showed that B-lymphocytes contribute to AHR, by using B-KO mice in a cockroach-induced asthma model. They were the first to provide evidence that antigen presentation by B-lymphocytes contributes to the pathogenesis of allergic disease [2]. It has also been suggested that B-lymphocytes may become increasingly relevant as antigen presenting cells when antigen load is low [10]. Our data confirmed the expression of MHCII and co-stimulatory molecules on the surface of B-lymphocytes of TDI-sensitized mice thus suggesting a role of antigen presentation for B-lymphocytes. Furthermore, there are several subsets of mature B-lymphocytes in the mouse. There are B2-lymphocytes (follicular and marginal zone B-lymphocytes), which arise from bone marrow derived precursors and are enriched in secondary lymphoid organs; on the other hand there are also B1-lymphocytes (B1a and B1b lymphocytes), which arise from fetal liver precursors and are enriched in mucosal tissues and the pleural and peritoneal cavities [31]. Follicular B-lymphocytes participate in the vast majority of responses against exogenous antigens, while marginal zone and B1-lymphocytes are characterized by their contribution to innate-like defense through rapid humoral responses [32]. We found in the auricular lymph nodes of TDI-sensitized mice significant increases in follicular B-lymphocytes as well as B1-lymphocytes, indicating that both subsets are probably important in the allergic response we find.

The knowledge that CD4^+^ T-lymphocytes can produce polarized arrays of cytokines has been extended over the last years to include CD8^+^ T-lymphocytes, natural killer cells and dendritic cells. It is also known that B-lymphocytes are major producers of a broad range of cytokines, but it was not until recently that evidence was obtained that B-lymphocytes can be induced to differentiate into distinct cytokine producing effector subsets [11,23]. Harris et al. showed in an infection model that B-lymphocytes have the capacity to produce cytokines such as IL-2, IFN-γ, IL-12 and IL-4, which have not been traditionally considered to be B-lymphocyte derived cytokines [11]. B-lymphocytes of TDI-sensitized mice produced *in vitro* substantial amounts of IL-4, IFN-γ or IL-10, suggesting the presence of Be2 lymphocytes as well as Be1 lymphocytes in our mouse model. TDI sensitization yields a mixed Th1-Th2 cytokine profile, as previously described by us and other research groups [15,16,19,33,34]. Our present results show that probably the same is true for B-lymphocytes. The mixed cytokine profiles found in chemical-induced asthma are in contrast with the Th2 prone response found in atopic asthma, and make it challenging to understand how the development of this type of asthma is regulated.

To strengthen our results, the adoptive transfer experiments were repeated in B-KO mice. When we applied our classic model of dermal sensitization followed by a single airway challenge with TDI, no asthma-like response was found in B-KO mice, but this response could be regained after the transfer of B-lymphocytes. Again, we found no increases in total serum IgE levels in the B-KO mice that received B-lymphocytes. This leads us to the conclusion that IgE probably does not play predominant role in these experiments. Since B-KO mice still possess T-lymphocytes, and we could not exclude an interplay between these T-lymphocytes and the transferred B-lymphocytes, we also performed transfer experiments in SCID mice which lack both B- and T-lymphocytes. This resulted also in the induction of an asthma-like response. Apparently, B-lymphocytes do not need T-lymphocytes to initiate AHR and airway inflammation in mice. Our study is the first to prove that B-lymphocytes can solely lead to the development of an asthma-like response. In isocyanate-induced asthma the importance of CD4^+^ and CD8^+^ T-lymphocytes was already shown [34,35]. Our study does not imply that B-lymphocytes do not need T-lymphocytes or other cell types of the immune system to activate and differentiate during the sensitization phase, but it does suggest that T-lymphocytes are not exclusively needed for the effector phase in our model. Although, B-KO mice have defects in the homeostasis of the immune system, including fewer T-lymphocytes [25], we are convinced that the results of the transfer experiments in the B-KO mice can be interpreted as resulting essentially from their lack of B-lymphocytes rather than their defective T-lymphocytes because of the asthma-like responses we obtained in SCID mice receiving B-lymphocytes.

In conclusion, we have shown that B-lymphocytes play a crucial role in the development of an asthma-like response in a mouse model of chemical-induced asthma. Sensitization with TDI led to a mixed Be1-Be2 cytokine response and transferring these “sensitized” B-lymphocytes into naïve mice resulted in AHR and airway inflammation after challenge with TDI. Furthermore, the generation of a response in SCID mice suggests that B-lymphocytes can induce an asthmatic response without the help of T-lymphocytes.
